# Traumatic odontoid process synchondrosis fracture with atlantoaxial instability in a calf: clinical presentation and imaging findings

**DOI:** 10.1186/s13620-015-0034-5

**Published:** 2015-04-24

**Authors:** Velia-Isabel Hülsmeyer, Katharina Flatz, Katrin Putschbach, Martina Ramona Bechter, Sebastian Weiler, Andrea Fischer, Melanie Feist

**Affiliations:** Section of Neurology, Clinic of Small Animal Medicine at the Centre for Clinical Veterinary Medicine, Ludwig Maximilian University, Veterinärstr.13, 80539 Munich, Germany; Clinic of Small Animal Surgery and Reproduction at the Centre for Clinical Veterinary Medicine, Ludwig Maximilian University, Munich, Germany; Clinic for Ruminants with Ambulatory and Herd Health Services at the Centre of Clinical Veterinary Medicine, Ludwig Maximilian University, Munich, Germany

**Keywords:** Atlantoaxial instability, Tetraparesis, Dens axis, Bovine, Calf, Odontoid process

## Abstract

A 6-week-old female Simmental calf was evaluated for acute non-ambulatory tetraparesis. Physical and laboratory examinations revealed no clinically relevant abnormalities. Neurological findings were consistent with acute, progressive and painful cervical myelopathy. Radiographs displayed a fractured odontoid process (dens axis) and vertebral step misalignment at the fracture site. A traumatic origin was suspected. Advanced diagnostic imaging was considered to allow better planning of potential surgical stabilisation and to exclude any additional lesions of the cervical vertebral column. However, during trailer transportation to the advanced diagnostic imaging and surgery site, the calf deteriorated neurologically and was humanely euthanised. Magnetic resonance imaging (MRI) and computed tomography (CT) were performed immediately post-mortem for scientific reasons. The MRI examination reflected the radiographic findings and confirmed severe spinal cord compression at the fracture site. In addition, a T2W-hyperintense signal change within the paravertebral soft tissue dorsal to the fracture site was indicative of a traumatic event. CT identified the fracture site at the synchondrosis between the odontoid process and the body of the axis, and this finding was confirmed by post-mortem examination. Advanced diagnostic imaging and post-mortem examination did not identify any other cervical lesion. In summary, this calf was diagnosed with a traumatic odontoid process synchondrosis fracture, which has not been reported previously in calves but presents a challenging and well-known fracture type in young children. This case report indicates that the odontoid process synchondrosis is a potential predisposed injury site and that traumatic odontoid process synchondrosis fractures should be considered as a potential differential in calves with acute cervical pain and/or signs of a cervical myelopathy.

## Background

Atlantoaxial instability is a well-recognised neurological condition in humans and in small companion animals. It can manifest with a wide range of neurological signs varying from pain only to tetraplegia or even death. In small animals and in humans, atlantoaxial instability usually is caused by a traumatic event or a congenital malformation, such as odontoid process hypo- or aplasia [1-3]. Frequently, there also might be a combination of both, as minor traumas often provoke the acute onset of neurological signs in patients with congenital atlantoaxial malformations [4]. In addition to humans and small animals, atlantoaxial instability also has been reported in large animals, such as equines and ruminants [5-16]. However, in all previously reported bovine cases, the atlantoaxial instability was caused by a congenital malformation of the odontoid process (aplasia or hypoplasia) and/or atlantooccipital region (such as atlantooccipital fusion) [5-7]. Particularly, atlantooccipital fusion has been reported to promote secondary atlantoaxial instability even in the absence of odontoid process malformations [5]. Most previous bovine reports of atlantoaxial instability established their diagnosis by plane radiographs and/or necropsy [5-7]. However, one case series diagnosed congenital odontoid process abnormalities with subsequent atlantoaxial instability in five cattle by radiography and necropsy but identified concurrent atlantooccipital fusion only on necropsy in the majority of cases [7]. Very few reports of truly traumatic injuries of the first cervical vertebrae in cattle exist, such as one published case of a traumatic atlantooccipital dislocation in a calf [17] or a compression fracture of the second and third cervical vertebrae in a heifer [18]. However, there are no reports of traumatic odontoid process fractures in cattle. This case report is the first documentation of atlantoaxial instability caused by a traumatic odontoid process synchondrosis fracture in a calf diagnosed by radiography and further characterised by computed tomography (CT) and magnetic resonance imaging (MRI). Moreover, this manuscript gives an overview of current odontoid process fracture classification systems established in adults and young children, with the latter (classification of odontoid process synchondrosis fractures) having not yet been applied to veterinary medicine.

## Case presentation

**Figure 1:**
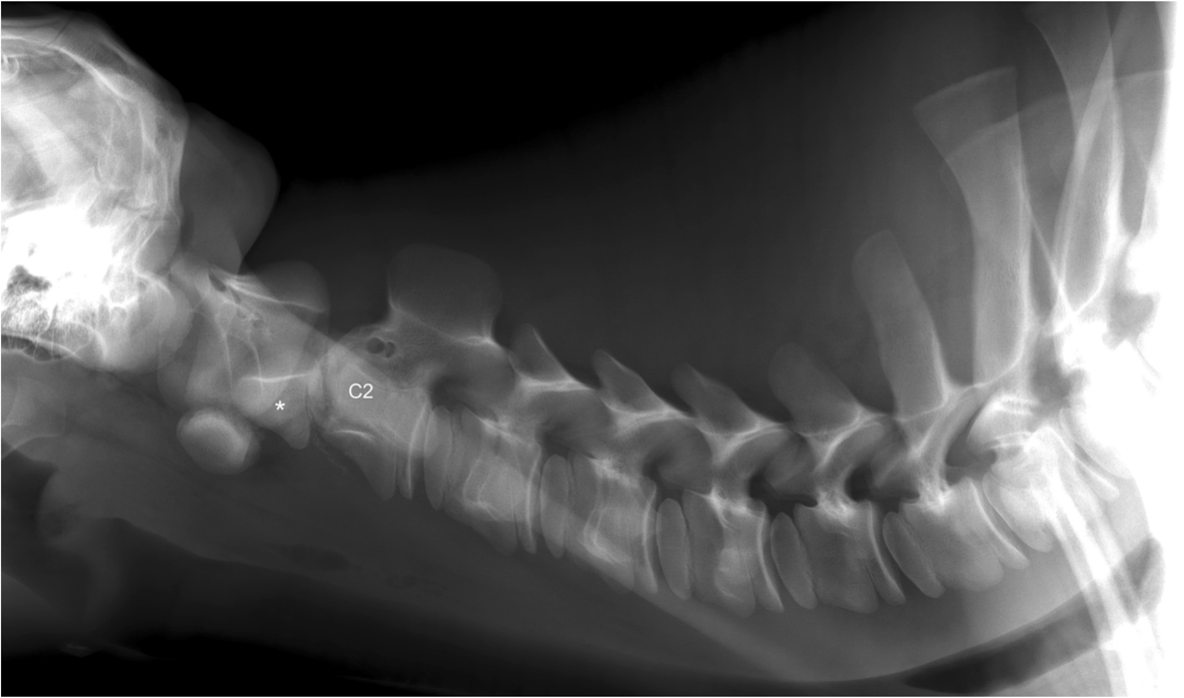
Laterolateral digital radiograph of the cervical vertebral column. The odontoid process (asterisk) is fractured and displaced but still remains attached to the body of the atlas. Notice the marked step misalignment between the fracture fragments and narrowing of the vertebral canal at this level.

**Figure 2:**
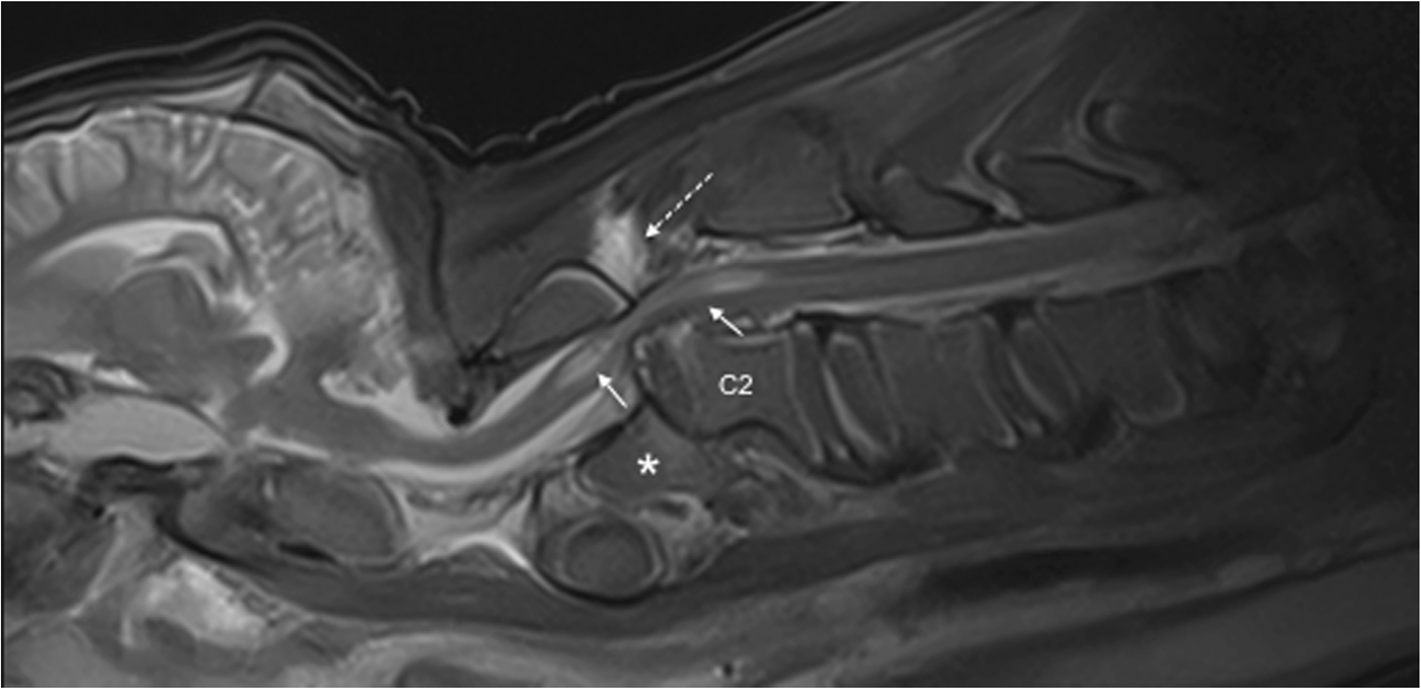
Post-mortem sagittal T2W-image of the cervical vertebral column. The MRI revealed similar findings as the radiographs but displayed pronounced spinal cord involvement. The images reflect displacement of the C2 vertebral body with step misalignment of the vertebral canal and severe spinal cord compression at this level. Next to the compression site, the spinal cord (continuous arrows) shows a T2-hyperintense intramedullary signal. The considered differentials were spinal cord oedema, haemorrhage or myelomalacia. The T2-hyperintense area within the paravertebral musculature located dorsal to the fracture site (broken arrow) is indicative of a traumatic dorsal event. Asterisk = odontoid process.

**Figure 3:**
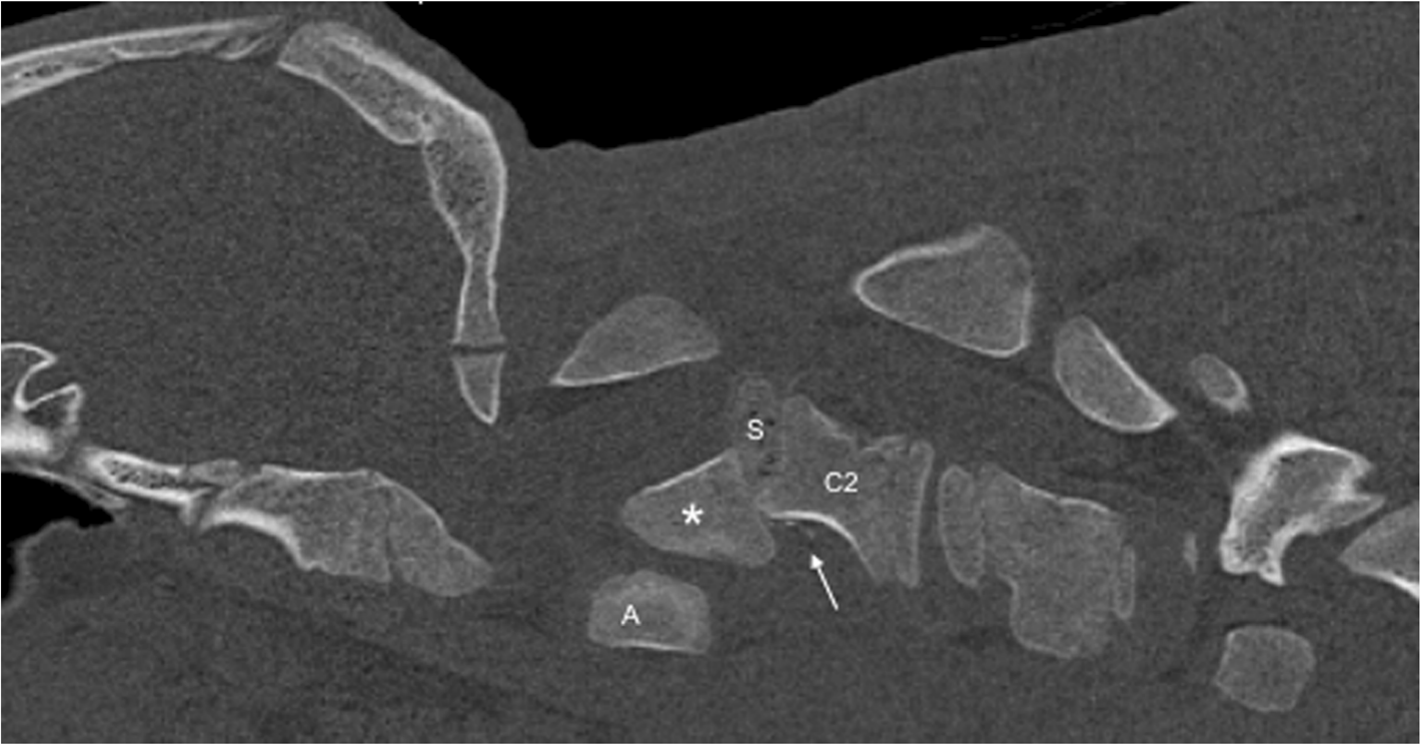
The fractured but normally developed odontoid process (asterisk) is highlighted by sagittal reconstruction of the post-mortem CT examination. The fracture site is located at the synchondrosis (S) between the base of the odontoid process and the body of the axis (C2). Comminuted bony fragments are visible ventrally in this aspect (arrow), and the odontoid process displacement was calculated to be 40% (see the calculation formula in the text). A = atlas.

A 6-week-old female Simmental calf was found in lateral recumbency (igloo system, housed with one other calf). The non-ambulatory and recumbent calf was pre-treated by the referring veterinarian with metamizole and then referred to the Clinic for Ruminants for further evaluation. The physical and laboratory examinations (CBC, serum biochemistry profile and blood gases) showed no clinically relevant abnormalities. On neurological examination, the calf appeared conscious and responsive to stimuli but presented in lateral recumbency with non-ambulatory tetraparesis (minimal voluntary movements of all four limbs were preserved). Cranial nerve function was normal. With support, the calf could be raised but showed reduced to absent postural reactions in all limbs. Spinal reflexes were all normal, with increased muscle tone. Conscious pain perception was evaluated to be preserved. Pronounced upper cervical hyperaesthesia with intermittent opisthotonus was evident on neck palpation. Based on neurological findings, the lesion was localised to the C1-C5 spinal cord segments. Based on the clinical history and the calf’s age, the most likely differentials were considered to be trauma, anomaly or inflammation. Lateral cervical digital radiographs (Axiom Luminos dRF, Siemens Healthcare, Erlangen, Germany KV: 69,8, mAS: 25), identified a fractured odontoid process, which appeared to be still attached to the body of the atlas. However, dorsal dislocation of the C2 vertebral body was obvious (Figure 1). The remaining cervical vertebrae displayed no obvious radiographic abnormalities. Based on the radiographic findings our initial diagnosis was a traumatic odontoid process fracture. Instability and spinal cord compression at the fracture site were highly suspected due to the radiographic findings and neurological presentation. Therefore, surgical decompression, anatomical alignment, and stabilisation were favoured for our calf rather than conservative management (such as neck splinting). Potential surgical stabilisation was planned to be performed in collaboration with our small animal neurosurgeons located at the Clinic for Small Animal Surgery and Reproduction. Although the diagnosis of an odontoid process fracture was already established on radiographs, advanced diagnostic imaging was considered to allow better surgical planning and to screen for other traumatic lesions within the remaining cervical vertebrae (because it is reported that multiple vertebral lesions may occur in 5-10% of small animal trauma patients [19,20]). In addition, the owner requested advanced diagnostic imaging examination prior to a cost-consuming surgery to definitively exclude any concurrent and injury-predisposing congenital abnormality at the atlantooccipital region. In the case of a concurrent congenital atlantooccipital fusion, the calf’s owner would have denied any further treatment due to the condition’s potential heritability and associated limitation of future breeding uses. However, because of rapidly deteriorating neurological signs and respiratory distress during trailer transportation to the advanced diagnostic imaging and surgery sites (both located at the Clinic for Small Animal Surgery and Reproduction), the calf was euthanised. MRI and CT examinations were performed immediately post-mortem for scientific reasons. The MRI examination was performed using a 1.5 T magnetic resonance unit (Magnetom Symphony, Siemens Healthcare, Erlangen, Germany). The dead calf was positioned in sternal recumbency because this allowed positioning with only minor neck manipulation (compared with dorsal recumbency) and hence was assumed to lower the risk of artificial and manipulation-induced fracture dislocation; however, dorsal recumbency would have allowed closer contact between the area of interest and the table coils. T1-weighted (T1W; TR: 768, TE: 13), T2-weighted (T2W; TR: 3980, TE: 109) and short tau inversion recovery (STIR; TR: 5790, TE: 61, TI: 140) pulse sequences of the cervical vertebral column were obtained in the sagittal (T1W, T2W and STIR) and dorsal planes (STIR) with a slice thickness of 3 mm (STIR) and 2.5 mm (T1W and T2W). The MRI examination confirmed severe spinal cord compression at the level of the fracture site due to dorsal displacement of the C2 vertebral body and pronounced vertebral step misalignment (Figure 2). Cranial and caudal to the compression site, the spinal cord appeared swollen and exhibited a hyperintense intramedullary signal on the T2W-images and on the STIR-images, with mixed intensity (but predominantly hypointense appearance) on the T1W-images. Based on signal-pattern spinal cord oedema, intramedullary haemorrhage or myelomalacia were considered as potential differentials. A T2* gradient echo (T2*-GRE) sequence would have been helpful to further characterise this spinal cord T2W-hyperintensity but unfortunately was not performed. Dorsal to the compression site, the paravertebral musculature showed a focal area of T2-hyperintensity (with similar appearance on the STIR-images), which was supposed to be indicative of a dorsal traumatic event at this level. A post-mortem CT examination of the cervical vertebral column was performed with a 64-slice, helical CT scanner (Somatom Definition AS, Siemens Healthcare, Erlangen, Germany). Transverse images of the cervical vertebral column were acquired (64 x 0.6-mm detector collimation, 120 KVp, 120 ma, 1 sec rotation time and 512 x 512 reconstructed image matrix). A bone algorithm (B70s convolution Kernel) was used for reconstruction. CT images reflected MRI findings and furthermore identified the fracture site at the synchondrosis between the body of the axis and the odontoid process (Figure 3). The fracture line was directed in a dorsoventral oblique direction from the dorsal area of the odontoid process to the ventral aspect of the body of the axis. Additional findings were bony fragments at the ventral aspect of the fracture line. The degree of odontoid process displacement was calculated to be approximately 40%, employing the following formula used in young children with odontoid process synchondrosis fractures: Distance of maximum displacement of the fractured odontoid process (distance from the cortex of the odontoid to the outer cortex of the body of C2) divided by the anteroposterior diameter (equals the dorsoventral diameter in animals) of the odontoid [21]. Potential congenital malformations predisposing to atlantoaxial instability and other traumatic lesions of the remaining vertebrae were definitely ruled out by advanced diagnostic imaging examination and subsequent necropsy. In summary, this calf was diagnosed with a traumatic odontoid process synchondrosis fracture.

## Discussion and conclusion

This is the first case report of a traumatic odontoid process synchondrosis fracture in a calf. Neurological examination was useful for early and correct neuroanatomical localisation. Radiography was easy to perform and allowed an initial diagnosis. MRI and CT examination (although performed post-mortem for scientific interest) were helpful for sub-classifying the fracture type, excluding additional (traumatic or congenital) cervical lesions and, potentially, allowing better planning of potential surgical interventions. However, as evident in our case, transportation to the advanced diagnostic imaging site incurred the risk of further displacement and neurological deterioration, suggesting that the benefits versus risks of any procedure and transport should be carefully considered.

So far, detailed classification systems of atlantoaxial fractures are lacking in veterinary medicine. In humans, several classification systems for odontoid process fractures have been established but are still a process under progress. The most popular system is the Anderson & D’Alonzo classification system, which describes three different fracture types: Type I fractures affect only the tip of the odontoid process, type II fractures are located at the base of the odontoid process, and type III fractures extend into the body of the axis [3]. In recent years, type II fractures (all located at the base of the odontoid process) have been further divided into three different subclasses by several authors. Subclass IIA is defined by a non-displaced horizontal fracture, subclass IIB consists of anterior superior to posterior inferior fractures (based on sagittal views in people) or displaced transverse fractures, and subclass IIC is classified by anterior inferior to posterior superior or comminuted fractures [22]. However, one recent study specifically focussed on odontoid process fractures in young children with an open synchondrosis and established a new classification system with fractures located at the synchondrosis (type I) and fractures located proximal to the synchondrosis (type II) [21]. Type I fractures are further sub-classified based on the degree of odontoid process displacement in type IA (displacement < 10%), type IB (displacement 10-100%) and type IC (displacement > 100%) [21]. Applying the latter classification system used in young children, a type IB odontoid process fracture was most compatible with the imaging findings in our calf (fracture at the synchondrosis with an odontoid process displacement of 40%).

In humans, fractures affecting the base of the odontoid process represent the most common fracture type among odontoid process fractures, with a bimodal age distribution (young children and elderly adults) [3,23]. This may be attributed to the unique anatomy and architecture of the base of the odontoid process, predisposing it as an area of potential weakness prone to injury following cervical traumas [24-27]. In elderly patients, the base of the odontoid process is distinguished from other areas in the axis by a lower bone density as well as a decreased trabecular interconnection [27,28], whereas in young children, the axis is divided by several synchondroses [26]. The synchondrosis between the base of the odontoid process and the body of the axis does not fully ossify until a child is six to seven years of age and hence is particularly prone to traumatic injury [26]. In foals, the same synchondrosis closes by the age of seven to nine months [29], and similar to the case in children, centres of ossification in foals are reported to be unable to withstand excessive pulling forces [30]. An ossification study of the prenatal and neonatal bovine skeleton revealed that the ossification centre of the odontoid process was present at an estimated foetal age of 80 days, but complete ossification did not form prenatally or by one month of age [31]. Consequently, in young calves – similar to the case in young children – the synchondrosis of the odontoid process should be considered as an area of potential weakness, which may be prone to injury at least during the first month of life.

In children, fractures at the odontoid process synchondrosis are often secondary to high-energy traumas by motor vehicle accidents (due to hyperflexion/hyperextension of the upper neck), whereas in elderly patients fractures of the odontoid process mainly appear as a result of low-energy injuries (such as falling from standing height) [32-35]. In small animals, odontoid process fractures commonly result from an animal running at a high speed into an obstacle, during which the neck is forcibly flexed [20]. Similarly, traumatic hyperflexion or hyperextension of the neck (e.g. during falling), as well as accidents during haltering, are reported to cause atlantoaxial fractures in horses [13,36]. Unfortunately, the exact trauma origin in our bovine case remains unclear due to lack of an observed traumatic event. However, conceivable cervical trauma sources in calves may potentially be attributed to traumatic hyperextension or hyperflexion of the neck by accidents in feeding grids or cattle panels, by calf tethering or by incorrectly assisted parturition. A T2W-hyperintense signal within the paravertebral musculature at the level of the fracture site was identified in our patient and has been described in dogs with traumatic spinal fractures [37]. Hence, this finding further supported our diagnosis of a traumatic origin.

The ideal treatment of odontoid process fractures in humans (e.g., surgical versus non-surgical management) still remains a matter of debate [38]; however, in displaced fractures, surgical stabilisation is usually the treatment of choice. Additionally, the severity of neurological signs often affects the decision-making process between surgical stabilisation and conservative management. Although successful conservative management (splinting of the neck) has been reported in horses with atlantoaxial instability [13,39], this was not considered to be a reasonable option for our bovine patient given the radiographically visible narrowing of the vertebral canal and the pronounced neurological signs. However, as pain perception and minimal voluntary movements were present on initial neurological examination, surgical stabilisation and decompression were considered. Successful surgical atlantoaxial stabilisation has been reported only once in a calf [5] but several times in horses [12,14,16,40] and is a well-established procedure in dogs [4]. Surgery was planned to be conducted in close collaboration with our small-animal surgeons at the Clinic for Small Animal Surgery and Reproduction due to their expertise with spinal surgeries and the availability of the equipment required for spinal stabilisation. Although radiography facilitated an initial diagnosis, we decided (in accordance with the owner) to perform advanced diagnostic imaging examination prior to surgery to allow better planning of the surgery and to disclose any other concurrent cervical lesions (traumatic or congenital). However, the calf was euthanised during transport to the imaging and surgery sites; hence, no surgical intervention was performed, and advanced diagnostic imaging was conducted post-mortem only for scientific interest, without any clinical relevance.

The limitations of this case report are the negative impacts of transport and positioning for advanced diagnostic imaging. The neurologic signs worsened during transport; therefore, we cannot exclude that further vertebral displacement, spinal cord injury or even myelomalacia occurred. However, trailer-transportation to the surgery and advanced diagnostic imaging sites was essential in our case because we considered surgery to be superior to conservative management. Yet, any procedure and transport should be carefully considered based on a risk-benefit calculation and case-by-case analysis. One major limitation of the MRI examination is the not-performed T2*-GRE sequence, which would have been useful to better define the identified intramedullary spinal cord changes. A signal void on T2*-GRE might have allowed a diagnosis of intramedullary haemorrhage [37]; however, this finding might also have been compatible with haemorrhagic myelomalacia [41].

In summary, this report is unique because a traumatic odontoid process synchondrosis fracture in a calf has not been described previously in the veterinary literature. Applying the classification system for odontoid process synchondrosis fractures used in young children, we diagnosed a type IB odontoid process synchondrosis fracture. Odontoid process synchondrosis fractures should be considered as a potential differential in calves with neck pain and/or signs of C1-C5 myelopathy, and current odontoid process synchondrosis fracture classification systems used in young children are applicable to veterinary patients.

## Abbreviations

MRI: Magnet resonance imaging
CT: Computed tomography
T1W: T1-weighted
T2W: T2-weighted
STIR: Short tau inversion recovery
T2*-GRE: T2*-gradient echo
